# Reflectance Confocal Microscopy in Evaluating Skin Cancer: A Clinicians's Perspective

**DOI:** 10.3389/fonc.2019.01457

**Published:** 2019-12-19

**Authors:** Angela Filoni, Mauro Alaibac

**Affiliations:** ^1^Melanoma and Soft Tissue Sarcoma Unit, Veneto Institute of Oncology, IOV-IRCCS, Padua, Italy; ^2^Unit of Dermatology, University of Padua, Padua, Italy

**Keywords:** reflectance confocal microscopy, dermatology, histopathology, skin cancer, non-invasive skin imaging, diagnosis

Reflectance confocal microscopy (RCM) is a optical imaging technique that affords an horizontal view of the skin until superficial dermis. RCM uses a laser light, at near infrared wavelength (830 nm), as a source of coherent monochromatic light which penetrates the tissue and illuminates a single point.

Microscopic tissue elements reflect light with different refractive indices resulting in white structures on a black background. Given the high reflective index of melanin and keratin this non-invasive imaging tool can have an important application in the diagnosis of skin cancer. Though an “optical biopsy” allows physicians to visualize the skin lesion to a depth of 200 microns with subcellular resolution, improving diagnostic accuracy of dermoscopic equivocal cutaneous lesions (1).

If combined with clinical and dermoscopic diagnosis, RCM can enhance the accuracy of differentiating between benign and malignant skin neoplasms (2). RCM can improve early malignant melanoma diagnosis and can be a valuable method for the monitoring of skin lesions over time, reducing the number of unnecessary excisions, in particular in cases of cosmetically important areas, such as the face or the neck.

Despite its utility in skin cancer diagnosis the diffusion of RCM in clinical practice is circumscribed mainly around academic medical centers. Moreover, the daily use of RCM is limited by its cost and knowledge gap amongst dermatologist (3).

Before performing RCM at a safe level physicians need a dedicated training, that is not available although the numerous publications in literature about this topic (4).

Farnetani et al. (5) showed that accuracy in skin cancer recognition correlated directly with the expertise of the evaluator; as a matter of fact experienced RCM users had higher sensitivity than more novice RCM users (91.0 vs. 84.8%).

In RCM the images are in gray scale and the interpretation of RCM skin images requires specific knowledge of histomorphological features rather than direct translation of clinical and dermoscopic skills. Consequently, this makes RCM difficult to perform in a routine clinical setting.

Unlike dermoscopy, which has rapidly spread as a screening technique among dermatologists of all generations, currently RCM is rarely used outside teaching hospitals. Dermoscopy is based on the description of colors and structures, whereas RCM morphologic features are similar to those observed within a diagnostic pathology setting (Figure 1). Moreover, RCM-specific vocabulary and architectural features overlap with the diagnostic terminology used in histopathology that is different from that of clinical dermatology, for example cells types are identified through their morphology (polygonal, round, etc.) or their distribution pattern (nest, clustered, pagetoid) (6).

**Figure 1:**
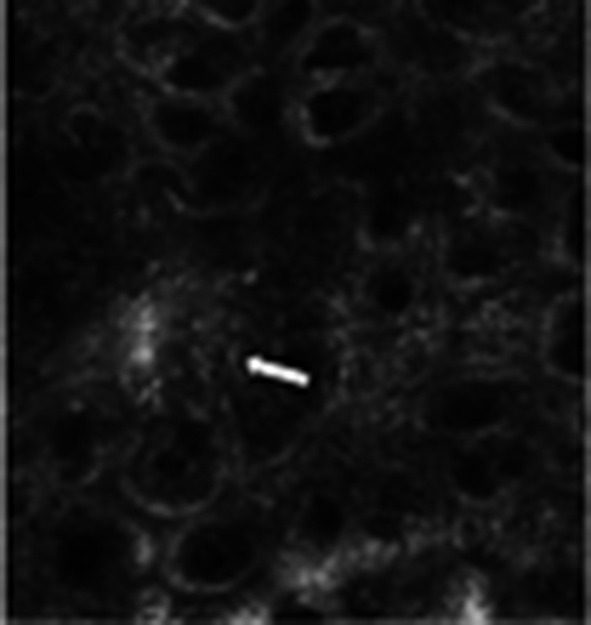
Lesion of the right leg of a 43 years old woman; histopathologic analysis revealed melanoma, focally invasive with a Breslow thickness of 0.41 mm. Confocal examination (Vivascope^®^ 1500) shows papillae that are not always well-defined and the presence of some dendritic pagetoid cells (arrow). This image indicates how the diagnosis is based on the histomorphology of the skin tissue and not on the clinical features of the lesion.

Also images visualization is more pertinent to the dermatopathology since in RCM there is an acquisition both in planes oriented parallel to the skin surface and both in depth through stack of images.

The above considerations indicate that RCM, in our opinion, may reach more widespread acceptance and utilization among pathologists/dermatopathologists rather than clinical dermatologists. To this regard, a recent article (7) reports a case series where prebiopsy examination with RCM improved the reliability of histopathological diagnosis allowing for a critical appraisal of initial histopathological misdiagnosis. The pathologist also plays a key role in a new emerging imaging technique that this is *ex vivo* confocal microscopy; this new technique allows real-time microscopic examination of freshly excised cutaneous tissues. This technology has been introduced in Mohs dermatologic surgery in particular for the evaluation of the surgical margins of basal cell carcinomas but shows a possible application in breast and brain surgery. *Ex vivo* confocal microscopy could save two-thirds of processing time compared with conventional analysis of Mohs surgery, as processing and sectioning times are eliminated. Moreover, the procedure does not impair further histopathology evaluation on frozen or paraffin sections.

In conclusion, we believe that both *in vivo* and *ex vivo* RCM should primarily be used to assist pathologists/dermatopathologists in the classical histopatological and immunohistochemical diagnosis of skin cancer and, consequently, should not be considered a clinical tool for dermatologists.

## Author Contributions

AF and MA wrote the manuscript. The final version of the manuscript was approved by all coauthors.

### Conflict of Interest

The authors declare that the research was conducted in the absence of any commercial or financial relationships that could be construed as a potential conflict of interest.

## References

[1] Ahlgrimm-Siess V Laimer M Rabinovitz HS Oliviero M Hofmann-Wellenhof R Marghoob AA. Confocal microscopy in skin cancer. Curr Dermatol Rep. (2018) 7:105–18. 10.1007/s13671-018-0218-9 29780659 PMC5956038

[2] Farnetani F Manfredini M Chester J Ciardo S Gonzalez S Pellacani G. Reflectance confocal microscopy in the diagnosis of pigmented macules of the face: differential diagnosis and margin definition. Photochem Photobiol Sci. (2019) 18:963–9. 10.1039/C8PP00525G 30938378

[3] Pellacani G Scope A Gonzalez S Guitera P Farnetani F Malvehy J. Reflectance confocal microscopy made easy: the 4 must-know key features for the diagnosis of melanoma and nonmelanoma skin cancers. J Am Acad Dermatol. (2019) 81:520–6. 10.1016/j.jaad.2019.03.085 30954581

[4] Witkowski A Łudzik J Soyer HP Telediagnosis with confocal microscopy: a reality or a dream? Dermatol Clin. (2016) 34:505–12. 10.1016/j.det.2016.05.013 27692456

[5] Farnetani F Scope A Braun RP Gonzalez S Guitera P Malvehy J. Skin cancer diagnosis with reflectance confocal microscopy: reproducibility of feature recognition and accuracy of diagnosis. JAMA Dermatol. (2015) 151:1075–80. 10.1001/jamadermatol.2015.0810 25993262

[6] Shahriari N Grant-Kels JM Rabinovitz H Oliviero M Scope A. *In vivo* reflectance confocal microscopy image interpretation for the dermatopathologist. J Cutan Pathol. (2018) 45:187–97. 10.1111/cup.13084 29178501

[7] Shahriari N Grant-Kels JM Rabinovitz H Oliviero M Scope A. Reflectance confocal microscopy may enhance the accuracy of histopathologic diagnosis: a case series. J Cutan Pathol. (2019) 46:830–8. 10.1111/cup.13548 31298761

